# Oral Hygiene and Dietary Behaviors Associated with Major Carious Lesions in Primary Dentition: A Cross-Sectional Study

**DOI:** 10.3390/children13070877

**Published:** 2026-06-30

**Authors:** Ștefania Alice Petrache, Ionela Teodora Dascălu, Mădălina Olteanu, Adina Monica Chiriac, Mihaela Ionescu, Ana Maria Rîcă, Lelia Mihaela Gheorghiță, Constantin Dăguci, Paula Perlea, Mihaela Jana Țuculină

**Affiliations:** 1 Doctoral School, University of Medicine and Pharmacy of Craiova, 200349 Craiova, Romania; 2 Department of Orthodontics, Faculty of Dental Medicine, University of Medicine and Pharmacy of Craiova, 200349 Craiova, Romania; 3 Department of Pedodontics, Faculty of Dental Medicine, University of Medicine and Pharmacy of Craiova, 200349 Craiova, Romania; adina.chiriac@umfcv.ro; 4 Department of Medical Informatics and Biostatistics, Faculty of Medicine, University of Medicine and Pharmacy of Craiova, 200349 Craiova, Romania; 5 Department of Endodontics, Faculty of Dental Medicine, University of Medicine and Pharmacy of Craiova, 200349 Craiova, Romania; 6 Department of Endodontics, Faculty of Dental Medicine, University of Medicine and Pharmacy Carol Davila of Bucharest, 050474 Bucharest, Romania

**Keywords:** pediatric dentistry, primary dentition, dental caries, parental knowledge, oral hygiene, toothbrushing, dietary habits, sweetened beverages

## Abstract

**Background/Objectives**: Dental caries in primary dentition is strongly related to daily oral hygiene routines and sugar-related dietary behavior. This study assessed oral hygiene and dietary behaviors associated with the clinical severity of carious lesions in primary dentition. **Methods**: This observational, cross-sectional, clinical-record-based study assessed 448 pediatric dental records from children examined in Craiova, Romania. After applying predefined eligibility criteria, 400 parent–child pairs were included. Children were aged 3–6 years and had primary or early mixed dentition with predominant primary teeth. Carious lesions were classified as absent, incipient, cavitated non-complicated caries, or advanced caries with pulpal/periapical involvement. The primary analysis focused on toothbrushing onset and frequency, toothbrush and toothpaste type, night feeding duration, sweets intake, and sweetened or carbonated beverage intake. Logistic regression was used to identify factors associated with higher odds of major carious lesions. Secondary clinical-record variables were analyzed exploratorily. **Results**: Carious lesions were recorded in 331 children, while 69 children were caries-free. Incipient caries, cavitated non-complicated caries, and advanced caries with pulpal/periapical involvement were recorded in 99, 116, and 116 children, respectively. Child age and sex were not significantly associated with caries severity. In the adjusted model, delayed toothbrushing onset (OR = 1.464; 95% CI: 1.148–1.866; *p* = 0.002) and sweetened beverage consumption (OR = 2.488; 95% CI: 1.561–3.963; *p* < 0.0005) remained independently associated with major carious lesions after Bonferroni correction. **Conclusions**: In this sample, major carious lesions in primary dentition were associated mainly with modifiable postnatal behaviors, particularly delayed initiation of toothbrushing and sweetened beverage intake. Due to the cross-sectional design, the findings should be interpreted as associations, not causal relationships.

## 1. Introduction

Dental caries remains one of the most common preventable oral diseases in children and continues to affect primary dentition worldwide. The World Health Organization estimates that untreated caries in deciduous teeth affects approximately 514 million children globally, with an estimated global prevalence of 43%. Primary teeth are vulnerable soon after eruption, and carious lesions may progress rapidly when plaque control, fluoride exposure, and dietary routines are inadequate. Untreated lesions in young children may cause pain, infection, impaired mastication, sleep disturbance, school absenteeism, reduced oral-health-related quality of life, and increased need for complex dental treatment [1,2].

Early childhood caries (ECC) is defined by the American Academy of Pediatric Dentistry as the presence of one or more decayed, missing due to caries, or filled tooth surfaces in any primary tooth in a child younger than 72 months. This definition emphasizes that caries in early childhood should not be interpreted only as a local tooth problem. ECC reflects the interaction between biological susceptibility, cariogenic biofilm, dietary exposure, fluoride availability, oral hygiene routines, family behavior, and social context. Enamel defects, early colonization with cariogenic bacteria, frequent intake of fermentable carbohydrates, insufficient plaque removal, limited access to preventive dental care, and socioeconomic vulnerability have all been described as relevant determinants of caries risk in young children [3,4,5].

Primary dentition has specific clinical relevance because it supports mastication, speech development, occlusal guidance, and normal eruption of permanent teeth. Severe carious lesions in primary teeth may therefore have consequences beyond the affected tooth. Advanced lesions may progress to pulpal involvement, abscess, fistula, pathological mobility, premature tooth loss, and emergency dental attendance. For this reason, caries severity is clinically important. A child with incipient enamel changes does not require the same level of intervention as a child with cavitated or advanced lesions with pulpal/periapical involvement. Distinguishing between absent, incipient, progressing, and advanced caries with pulpal/periapical involvement may help clinicians identify children who need intensified preventive counseling, closer follow-up, and earlier treatment planning [2,6].

Dietary behavior is a central component of caries development in primary dentition. Free sugars are metabolized by dental biofilm into acids that promote enamel demineralization. The risk increases when sugar exposure is frequent, prolonged, or occurs between meals. WHO recommends reducing free sugar intake to less than 10% of total energy intake, with further reduction below 5% providing additional benefit for dental caries prevention. In young children, sugar-sweetened beverages, sweetened teas, juices, sweet snacks, and bedtime intake of carbohydrate-containing liquids may contribute to sustained acidogenic episodes, especially when oral clearance is reduced during sleep [2,6,7,8].

Feeding habits during early childhood also require careful interpretation. Breastfeeding has recognized general health benefits and should not be presented as harmful in itself. However, prolonged nocturnal feeding after eruption of primary teeth, especially when combined with delayed toothbrushing, poor plaque control, low fluoride exposure, or additional dietary sugars, may increase the risk of caries development and progression. Therefore, feeding counseling in pediatric dentistry should be integrated with the timing of tooth eruption, bedtime routines, sugar exposure, fluoride use, and parental supervision of oral hygiene [9,10].

Preventive behavior must begin early because oral hygiene routines are usually established during the first years of life. Current pediatric oral health recommendations support the establishment of a dental home within six months after eruption of the first tooth and no later than 12 months of age. They also recommend twice-daily supervised toothbrushing with fluoridated toothpaste, using a smear or rice-sized amount for children younger than three years and a pea-sized amount for children aged three to six years. These recommendations are particularly relevant for preschool children, who cannot independently perform effective toothbrushing and depend on caregivers for daily plaque control, toothpaste selection, and dietary regulation [3,7,8].

Parental knowledge and parental behavior are therefore key determinants of oral health in young children. Parents and legal guardians influence when toothbrushing begins, how often it is performed, whether fluoridated toothpaste is used, how sugar-containing foods and drinks are offered, and when the child is taken to the dentist. However, knowledge does not always translate into practice. A caregiver may know that oral hygiene should start early but may still delay brushing, use non-fluoridated toothpaste, permit frequent sweets intake, or maintain nocturnal feeding habits for a prolonged period. This gap between information and daily practice is clinically important because caries prevention depends on repeated behaviors performed consistently in the home environment [9,10].

The source of parental oral health information may also influence preventive behavior. Dentists, family physicians, relatives, and combined sources can provide different levels of accuracy and practical guidance. In preschool children, dental professionals have a central role in translating general prevention messages into concrete instructions, including when brushing should begin, how much toothpaste should be used, which drinks should be avoided, how bedtime feeding should be managed, and when routine dental visits should be scheduled. In Romania, recent data have also shown disparities in parental knowledge, attitudes, and practices regarding ECC according to demographic variables and living environment, supporting the need for locally relevant clinical data [9,10].

Prenatal and early-life factors may contribute to oral health vulnerability, although their interpretation requires caution. Maternal smoking during pregnancy has been associated with childhood dental caries in systematic reviews, but this association may be influenced by socioeconomic status, parental education, dietary routines, oral hygiene behavior, and access to care. Evidence regarding maternal antibiotic exposure or systemic medication use during pregnancy and later dental outcomes is less consistent and depends on the type of medication, timing, dosage, indication, and outcome assessed. In retrospective clinical-record-based studies, such variables should therefore be interpreted as exploratory anamnestic indicators rather than as direct causal exposures [11,12].

Given the multifactorial nature of dental caries in primary dentition, clinical-statistical assessment should integrate oral hygiene onset, toothbrushing frequency, toothpaste type, feeding practices, sweets intake, sweetened beverage intake, parental information, and relevant prenatal or postnatal anamnestic variables. Such an approach may help distinguish behaviors associated with early lesions from those associated with progressing or advanced caries with pulpal/periapical involvement. Therefore, the present study primarily aimed to assess oral hygiene and dietary behaviors associated with the prevalence and clinical severity of carious lesions in primary dentition. Parental information level, prenatal nicotine exposure, maternal antibiotic or systemic medication use during pregnancy, and lactose intolerance were assessed as secondary exploratory variables. From a clinical diagnostic perspective, identifying behavioral profiles associated with major carious lesions may help clinicians recognize children who require closer preventive assessment during routine pediatric dental evaluation.

## 2. Materials and Methods

### 2.1. Study Design and Setting

This study was designed as an observational, cross-sectional, clinical-statistical study based exclusively on data extracted from the clinical records of pediatric dental patients. The study evaluated the association between parental oral health information, hygiene-related behavior, dietary habits, prenatal and postnatal anamnestic factors, and the severity of dental caries in primary dentition.

Data were collected from the clinical records of children examined between June 2023 and March 2025 in participating clinical units in Craiova, Romania. The study was conducted in accordance with the principles of the Declaration of Helsinki and was approved by the Ethics Committee of the University of Medicine and Pharmacy of Craiova, approval no. 123/15 June 2023.

All variables analyzed in the present study were retrieved from existing pediatric dental files and from the anamnestic and clinical information documented during routine dental assessment.

Reporting followed the STROBE recommendations for cross-sectional observational studies.

### 2.2. Data Source and Study Sample

A total of 448 pediatric dental clinical records were assessed for eligibility. After applying the predefined inclusion and exclusion criteria, 400 records were retained for the final analysis, corresponding to 400 parent–child pairs. Forty-eight records were excluded because they did not meet one or more eligibility criteria or contained incomplete or ambiguous information for the variables required in the study.

The included children were aged between 3 and 6 years and presented primary or early mixed dentition with predominant primary teeth at the time of clinical examination. For each child, data were extracted from the pediatric dental clinical file, including the dental examination record and the anamnestic information provided by the parent or legal guardian during routine clinical history taking.

The clinical files contained information regarding prenatal history, postnatal feeding habits, oral hygiene routines, dietary exposure, parental source of oral health information, and dental clinical status. Only records with complete anamnestic and clinical data for the variables included in the study were retained for analysis.

Child age at examination and sex were available in the extracted database and were included as demographic variables. Age was analyzed within the restricted 3–6-year interval of the study sample, and sex was analyzed according to the category recorded in the clinical file.

### 2.3. Eligibility Criteria

Clinical records were included if they met all of the following criteria:

- The file belonged to a pediatric patient with primary or early mixed dentition with predominant primary teeth;
- The file contained information regarding the child’s age at examination and sex;
- The child had undergone a clinical dental examination recorded in the file;
- The file contained complete data regarding the presence and severity of carious lesions;
- The file contained complete anamnestic information provided by the parent or legal guardian;
- The file contained information regarding oral hygiene onset, toothbrush type, toothpaste type, night feeding, sweets intake, sweetened beverage intake, and carbonated beverage intake;
- The file contained information regarding prenatal exposure variables, including maternal smoking during pregnancy and maternal antibiotic or systemic medication use during pregnancy;
- The file allowed classification of the parent or legal guardian according to the recorded level of oral health information.

Clinical records were excluded if one or more of the following criteria were present:

- Incomplete clinical or anamnestic data for the variables analyzed;
- Absence of a recorded clinical dental examination;
- Inability to classify caries status based on the clinical record;
- Incomplete information regarding oral hygiene or dietary variables;
- Incomplete information regarding prenatal exposure variables;
- Records of patients with permanent dentition if the clinical assessment no longer allowed evaluation of primary dentition;
- Records with extensive prior dental treatment that prevented reliable classification of baseline caries severity.
- Because the study was based on existing clinical records, missing or ambiguous entries were not imputed. Files with missing essential variables were excluded from the corresponding analysis.

### 2.4. Variables Extracted from Clinical Records

The extracted variables were grouped into four categories. Demographic variables included child age at clinical examination and sex. These variables were considered to determine whether caries severity differed according to basic demographic characteristics within the included 3–6-year age range.

The first category included oral hygiene-related variables: reported age at onset of toothbrushing, toothbrush type, and toothpaste type. The onset of toothbrushing was categorized as 4 months, 6 months, 12 months, or 24 months. Toothbrush type was categorized as manual or electric. Toothpaste type was categorized as fluoridated toothpaste, non-fluoridated toothpaste, or unknown fluoride content, which was grouped with non-fluoridated toothpaste for analysis and considered a potential source of exposure misclassification.

The second category included dietary and behavioral variables: duration of night feeding after 6 months of age, frequency of sweets intake, intake of sweetened beverages, and intake of carbonated beverages. The duration of night feeding was categorized as 1 year and 6 months, 2 years, 2 years and 6 months, 3 years, or 3 years and 6 months. Frequency of sweets intake was categorized as 2–3 times per day, once per day, several times per week, or rarely. Sweetened beverage intake and carbonated beverage intake were recorded as dichotomous variables.

The third category included sources of parental oral health information. These were recorded as dentist, family physician, parents or relatives, or combined sources.

Regarding prenatal exposure, the variable ‘Maternal antibiotic or systemic medication use during pregnancy’ was defined as a general, exploratory indicator encompassing any documented maternal use of antibiotics or other systemic medications. Due to the retrospective nature of the clinical-record-based data, specific pharmacological details—such as the exact trimester of exposure, dosage, duration, or specific class of the antibiotic—could not be systematically retrieved. Therefore, this variable was interpreted as an exploratory anamnestic variable and not as a specific pharmacological exposure.

Lactose intolerance was recorded as a clinical-record variable when it was documented in the pediatric file or in the anamnestic information provided by the parent or legal guardian. The available records did not provide systematic information on diagnostic criteria, age at diagnosis, symptom severity, milk substitutes, lactose-free products, added sugars, feeding frequency, or bedtime intake. Therefore, lactose intolerance was analyzed as an exploratory variable.

The fourth category included clinical dental status. Carious lesions were classified as absent, incipient caries, cavitated non-complicated caries, or advanced caries with pulpal/periapical involvement, according to the clinical information recorded in the dental file.

### 2.5. Definition of Parental Information Level

Parental information level was extracted from the clinical record based on the anamnestic information documented during the pediatric dental visit. Parents or legal guardians were classified as informed when the clinical file indicated correct knowledge regarding the recommended timing for initiating oral hygiene in relation to the eruption of the first primary teeth. Parents or legal guardians were classified as uninformed when the clinical file indicated delayed or incorrect knowledge regarding the initiation of oral hygiene.

Because only 13 parents were classified as uninformed, parental information level was retained as a secondary descriptive and exploratory variable. The variable was kept in the analysis because it describes an important clinical and educational context, but comparisons involving parental information level were not used as primary evidence for the main conclusions of the study.

Based on this criterion, 387 parents were classified as informed and 13 parents were classified as uninformed.

### 2.6. Clinical Dental Assessment and Caries Classification

Dental clinical status was extracted from the pediatric dental examination recorded in each clinical file. The assessment referred only to temporary teeth or to temporary teeth present in predominantly primary dentition. Permanent teeth, when present, were not included in the caries severity classification used for the present analysis.

Caries status was classified according to the most severe carious lesion recorded in the clinical file. The diagnostic categories used in the study were defined a priori and were clinically mapped to the severity logic of the International Caries Detection and Assessment System (ICDAS) [13]. However, they did not represent a full ICDAS examination, because caries classification was performed retrospectively using the diagnostic information available in clinical records.

All original clinical assessments were performed during routine pediatric dental care by the same dentist. Because this was a retrospective clinical-record-based study, the original examinations were not performed as part of a prospective ICDAS-calibrated research protocol. Therefore, no formal prospective ICDAS examiner calibration was performed. Consistency of classification was supported by the use of a single original clinical examiner and by the uniform retrospective application of predefined four-level case definitions to all eligible records. Classification was based on the recorded clinical description of the most severe lesion, including the presence or absence of cavitation, dentin involvement, signs or symptoms suggestive of pulpal or periapical involvement, abscess, fistula, pathological mobility related to infection, and documented indication for endodontic or extraction-oriented treatment due to caries. Records with insufficient or ambiguous clinical information for assigning caries severity were excluded.

The four clinical categories were defined as follows:

1. No caries: no recorded clinical evidence of active or inactive carious lesions in temporary teeth.
2. Incipient caries: early non-cavitated carious lesions, corresponding clinically to visible enamel changes without dentin exposure, cavitation, pulpal involvement, abscess, fistula, or indication for endodontic or extraction-oriented treatment. This category was consistent with early enamel lesions comparable to ICDAS codes 1–2.
3. Cavitated non-complicated caries: cavitated carious lesions or lesions with clinical progression into enamel or dentin, without clinical signs or symptoms suggestive of pulpal or periapical involvement. This category included lesions comparable to ICDAS codes 3–5 when no pulpal or periapical complication was recorded.
4. Advanced caries with pulpal/periapical involvement: advanced carious lesions associated with clinical signs or symptoms suggestive of pulpal or periapical involvement, including extensive coronal destruction, spontaneous or persistent pain reported in the clinical history, abscess, fistula, pathological mobility related to infection, or documented need for endodontic or extraction-oriented treatment due to caries. This category included advanced cavitated lesions comparable to ICDAS code 6 when accompanied by clinical evidence of complication.

When more than one lesion was present, the child was assigned to the category corresponding to the most severe lesion recorded. This approach was used because the main outcome of the study was child-level caries severity rather than tooth-level or surface-level caries experience.

Because the study was based on existing clinical records, no additional examinations, radiographs, or diagnostic procedures were performed exclusively for research purposes. Radiographic information, when present in the clinical file, was used only as supporting clinical information and not as an independent inclusion criterion. Records were excluded when the available clinical information was insufficient to classify the presence or severity of carious lesions.

For the logistic regression analysis, caries severity was dichotomized into two categories. The minor caries category included children with no caries, or incipient caries. The major caries category included children with cavitated non-complicated caries or advanced caries with pulpal/periapical involvement. This dichotomization was based on clinical severity: no caries and incipient lesions represented absent or early non-cavitated disease, whereas cavitated non-complicated caries and advanced caries with pulpal/periapical involvement represented clinically established or advanced lesions requiring more intensive management.

### 2.7. Data Extraction Procedure

Data extraction was performed by reviewing the clinical files of eligible pediatric patients. The variables of interest were entered into a centralized database for statistical analysis. Each record was checked for completeness before inclusion.

To reduce transcription errors, categorical variables were coded using predefined categories corresponding to the categories recorded in the clinical files. When the information in the file was unclear, incomplete, or insufficient for classification, the record was not included for that variable.

No direct patient identifiers were included in the statistical database. Each clinical file received a numerical code before analysis.

### 2.8. Bias and Data Quality Considerations

Because this was a clinical-record-based study, the quality of the analysis depended on the completeness and accuracy of the information documented in the patient files. Behavioral and dietary variables were originally reported by parents or legal guardians during clinical history taking and may therefore be affected by recall bias or social desirability bias.

The study relied exclusively on anamnestic and clinical data already recorded in pediatric dental files. This approach allowed for analysis of real-world clinical documentation, but limited the study to variables available in existing patient records.

The marked imbalance between the informed and uninformed parental groups was also considered when interpreting comparisons based on parental information level.

Age and sex were available and were tested in relation to caries severity. Neither variable showed statistical significance in this sample. However, the restricted age interval and the clinical-record-based design limit the interpretation of age- or sex-specific patterns. In addition, because the study relied on retrospective clinical records, caries classification depended on the diagnostic detail available in the original files. To reduce misclassification, only records containing sufficient clinical information to assign caries status to one of the predefined severity categories were retained.

Because the study used existing clinical records, no prospective ICDAS calibration could be performed. However, all original clinical examinations were conducted by the same dentist, which reduced inter-examiner variability. The caries severity categories should therefore be interpreted as retrospective clinical-record-based categories mapped to ICDAS severity logic, not as direct ICDAS scores.

### 2.9. Statistical Analysis

Data processing was carried out to determine the statistical significance of the observed differences between the study groups. The significance threshold *p* was used, where a *p* < 0.05 was considered statistically significant. Descriptive statistics of continuous variables were expressed as “mean ± standard deviation (SD)” or median, when the assumption of normality was not met, while nominal and ordinal parameters were expressed as frequency distributions and percentages. Statistical tests were applied with SPSS (Statistical Package for Social Sciences) software, version 26 (SPSS Inc., Armonk, NY, USA).

All continuous data series were analyzed for normality based on the Kolmogorov–Smirnov test. Based on these results, comparisons between groups for continuous or ordinal variables were performed using the Mann–Whitney U test, Jonckheere-Terpstra test, or Kruskal–Wallis H test. Associations were tested using Chi-square or Fisher’s Exact tests, as appropriate. The magnitude of the associations between categorical variables was reported using the index ω, computed as the square root of the chi-square value divided by the sample size. The effect size was considered very small for values < 0.1, small for values between 0.1 and 0.3, moderate for values between 0.3 and 0.5, and large for values > 0.5.

A binary logistic regression model was developed to evaluate the association between the studied parameters and the probability of having minor or major carious lesions. The primary analysis focused on oral hygiene-related and dietary variables associated with caries severity and with the odds of major carious lesions. Parental information level, prenatal nicotine exposure, maternal antibiotic or systemic medication use during pregnancy, and lactose intolerance were analyzed as secondary exploratory variables because they were not the main determinants retained in the final model and, for some variables, the available clinical records did not provide detailed contextual information. A Bonferroni correction was applied using all terms in the model, resulting in statistical significance being accepted when *p* < 0.00625.

## 3. Results

Of the 448 pediatric dental clinical records assessed for eligibility, 400 were included in the final analysis after applying the predefined inclusion and exclusion criteria. The final sample consisted of 400 parent–child pairs. All included children were aged between 3 and 6 years and had primary or early mixed dentition with predominant primary teeth at the time of examination. Child age and sex were analyzed as demographic variables in relation to caries severity. Neither variable showed a statistically significant association with caries severity in this sample. Therefore, the subsequent analyses were organized into a primary analysis focused on oral hygiene and dietary behaviors and secondary exploratory analyses focused on parental information level, prenatal variables, and lactose intolerance.

### 3.1. Study Sample and Caries Prevalence

Within the study group, only 17.25% of participants (69 children) had no dental caries at the time of examination. For the remaining children, carious lesions were categorized according to their severity: incipient caries (99 children, 24.75%), cavitated non-complicated caries (116 children, 29.00%), and advanced caries with pulpal/periapical involvement (116 children, 29.00%). Caries prevalence and severity were then analyzed according to the planned primary and secondary analyses.

### 3.2. Primary Analysis: Oral Hygiene and Dietary Behaviors Associated with Caries Severity

The associations between oral hygiene variables and caries severity are presented in Table 1.

Several hygiene-related variables were significantly associated with caries severity. Toothbrushing onset was statistically significantly associated with caries severity. The distribution of caries severity was relatively similar among children whose toothbrushing started at 4, 6, or 12 months. However, more than 40% of children whose teeth were brushed from the age of two years old have developed advanced caries with pulpal/periapical involvement, and only 5.75% had no caries at all. Median brushing starting time in the four types of caries groups were similar, and the distributions of brushing starting times were rather similarly shaped for all groups, as assessed by visual inspection of a histogram. It was hypothesized that there would be an increasing monotonic trend in brushing starting moments for increasing severity of caries. A Jonckheere-Terpstra test determined that there was a statistically significant increasing monotonic trend in brushing starting times, *p* = 0.038. Kendall’s τ_b_ between caries severity and brushing starting times was 0.088.

Daily toothbrushing frequency was also significantly associated with caries severity (*p* = 0.005). Children who brushed their teeth once per day had a higher proportion of advanced caries with pulpal/periapical involvement compared with those who brushed twice per day, whereas the caries-free category was more frequent among children with twice-daily brushing. The effect size was r = 0.181.

Toothbrush type was also associated with caries severity. Among children using electric toothbrushes, 38.89% had no caries and 33.33% had incipient caries, while 11.11% had advanced caries with pulpal/periapical involvement. Among children using manual toothbrushes, 16.23% had no caries, while cavitated non-complicated and advanced caries with pulpal/periapical involvement accounted for 29.58% and 29.84%, respectively. Overall, the differences between groups were statistically significant (*p* = 0.005).

For the entire study group, the use of fluoride toothpaste was similarly distributed between groups with various severities of caries. For children who used non-fluoridated toothpaste, there was a predominance of cavitated non-complicated caries (32.00%) and advanced caries with pulpal/periapical involvement (30.18%), while the percentage of children with no caries was the smallest (14.18%). Overall, the differences between groups were statistically significant (*p* = 0.025).

As indicated in Table 2, dietary and feeding-related variables were analyzed in relation to caries severity; parental information level is also shown in the table as a secondary exploratory variable.

Parental information level is reported in Table 2 as a secondary exploratory variable and is described in Section 3.3.1.

Night feeding duration was significantly associated with lesion severity. A Jonckheere-Terpstra test indicated a statistically significant monotonic trend between longer night feeding duration and increasing caries severity (*p* = 0.032). Kendall’s τb between caries severity and night feeding duration was 0.090.

Sweets intake frequency was also significantly associated with caries severity. A Jonckheere-Terpstra test indicated a statistically significant monotonic trend between sweets intake frequency and caries severity (*p* < 0.0005). Kendall’s τb between caries severity and sweets intake frequency was 0.208.

Sweetened beverage intake was also significantly associated with caries severity. On the other hand, carbonated beverages were not associated with caries’ severity.

The effect-size values indicated that the magnitude of these associations was generally low. Therefore, statistical significance in these comparisons should be interpreted together with effect-size values.

Night feeding duration had a low effect size (τb = 0.090), sweets intake frequency showed a low-to-moderate effect (τb = 0.208), and sweetened beverage intake showed a low-to-moderate effect (r = 0.234).

#### 3.2.1. Logistic Regression Analysis of Factors Associated with Major Carious Lesions

In previous analyses, dental caries severity was classified into four categories: absent, incipient caries, cavitated non-complicated caries, and advanced caries with pulpal/periapical involvement. For the logistic regression analysis, caries severity was dichotomized into minor caries, including absent and incipient lesions, and major caries, including cavitated non-complicated and advanced lesions with pulpal/periapical involvement. Thus, a binary logistic regression model was used to evaluate the association of oral hygiene and dietary factors with the likelihood of major carious lesions. No standardized residuals exceeded ±2.5. The logistic regression model was statistically significant, χ^2^(8) = 45.423, *p* < 0.0005. The model correctly classified 64.50% of cases. The sensitivity was 80.20%, the specificity was 42.90%, the positive predictive value was 66.00%, and the negative predictive value was 61.00%. Of the eight predictor variables included in the multivariable logistic regression model, only two remained statistically significant after applying the strict Bonferroni correction threshold (*p* < 0.00625): the toothbrushing onset (*p* = 0.002) and the consumption of sweetened beverages (*p* < 0.0005) (Table 3).

Variable coding is detailed in the footnote to Table 3. Later toothbrushing initiation was independently associated with higher odds of major carious lesions.

Under this conservative Bonferroni threshold (*p* < 0.00625), the daily frequency of toothbrushing (*p* = 0.036) and the frequency of sweets intake (*p* = 0.027) did not reach independent statistical significance. Similarly, the type of toothbrush (*p* = 0.064), toothpaste type (*p* = 0.916), night feeding duration (*p* = 0.811), and carbonated beverage consumption (*p* = 0.449) did not act as significant factors independently associated with higher odds of major carious lesions in this multivariable model.

A sensitivity logistic regression model including child age and sex was also performed. After adjustment for these demographic variables, the direction of the associations remained unchanged, and delayed toothbrushing onset and sweetened beverage intake remained the main predictors associated with higher odds of major carious lesions. Child age and sex did not materially change the estimates of the main behavioral predictors, suggesting no relevant confounding effect in this sample.

#### 3.2.2. Analysis of the Number of Caries

For each child, the number of incipient caries, cavitated non-complicated caries, and advanced caries with pulpal/periapical involvement was recorded in the medical files. The distribution by groups in relation to the number of various types of caries is indicated in Table 4.

The level of parents’ information was not significantly associated with the number of caries developed by children (*p* > 0.05). Similar results were obtained for the intake of fizzy/carbonated drinks, as well as for the two studied prenatal factors (exposure to nicotine, or exposure to antibiotics/other drugs administered during pregnancy), *p* > 0.05.

For incipient caries, night feeding duration was the only variable significantly associated with lesion number. Longer night feeding duration was associated with a higher median number of incipient lesions.

For cavitated non-complicated caries, several variables showed statistically significant associations. Night feeding duration was associated with the number of cavitated non-complicated lesions (*p* = 0.004). Lactose intolerance was also associated with the number of cavitated non-complicated caries (*p* = 0.016), and this finding was retained as a secondary exploratory observation.

The number of cavitated non-complicated caries was also associated with toothbrush type (*p* = 0.026), toothpaste type (*p* = 0.009), sweets intake frequency (*p* < 0.0005), and sweetened beverage intake (*p* < 0.0005).

For advanced caries with pulpal/periapical involvement, significant associations were observed with toothbrushing onset (*p* = 0.039), lactose intolerance (*p* = 0.001), daily toothbrushing frequency (*p* = 0.001), sweets intake frequency (*p* < 0.0005), and sweetened beverage intake (*p* = 0.005). Children with later toothbrushing onset, lower brushing frequency, frequent sweets intake, and sweetened beverage intake tended to present a higher number of advanced lesions with pulpal/periapical involvement.

The lactose intolerance association was considered exploratory because detailed diagnostic and dietary information was not available in the clinical records.

Carbonated beverage intake was not significantly associated with the number of incipient, cavitated non-complicated, or advanced caries with pulpal/periapical involvement. Significant associations with the number of lesions were observed for several postnatal variables, including night feeding duration, lactose intolerance, toothbrushing onset, daily toothbrushing frequency, sweets intake frequency, and sweetened beverage intake, depending on lesion type.

Prenatal nicotine exposure and maternal antibiotic or systemic medication use during pregnancy were evaluated as secondary exploratory variables. Their associations with lactose intolerance were reported descriptively only and were not used to support a biological or causal interpretation.

### 3.3. Secondary Exploratory Analyses: Parental Information Level, Prenatal Variables, and Lactose Intolerance

#### 3.3.1. Parental Information Level and Sources of Information

Of the 400 parents or legal guardians included in the analysis, 387 (96.8%) were classified as informed according to the clinical-record criterion used in this study, whereas 13 (3.2%) were classified as uninformed. Given the marked imbalance between these groups, parental information level was analyzed mainly as a descriptive variable.

**Figure 1:**
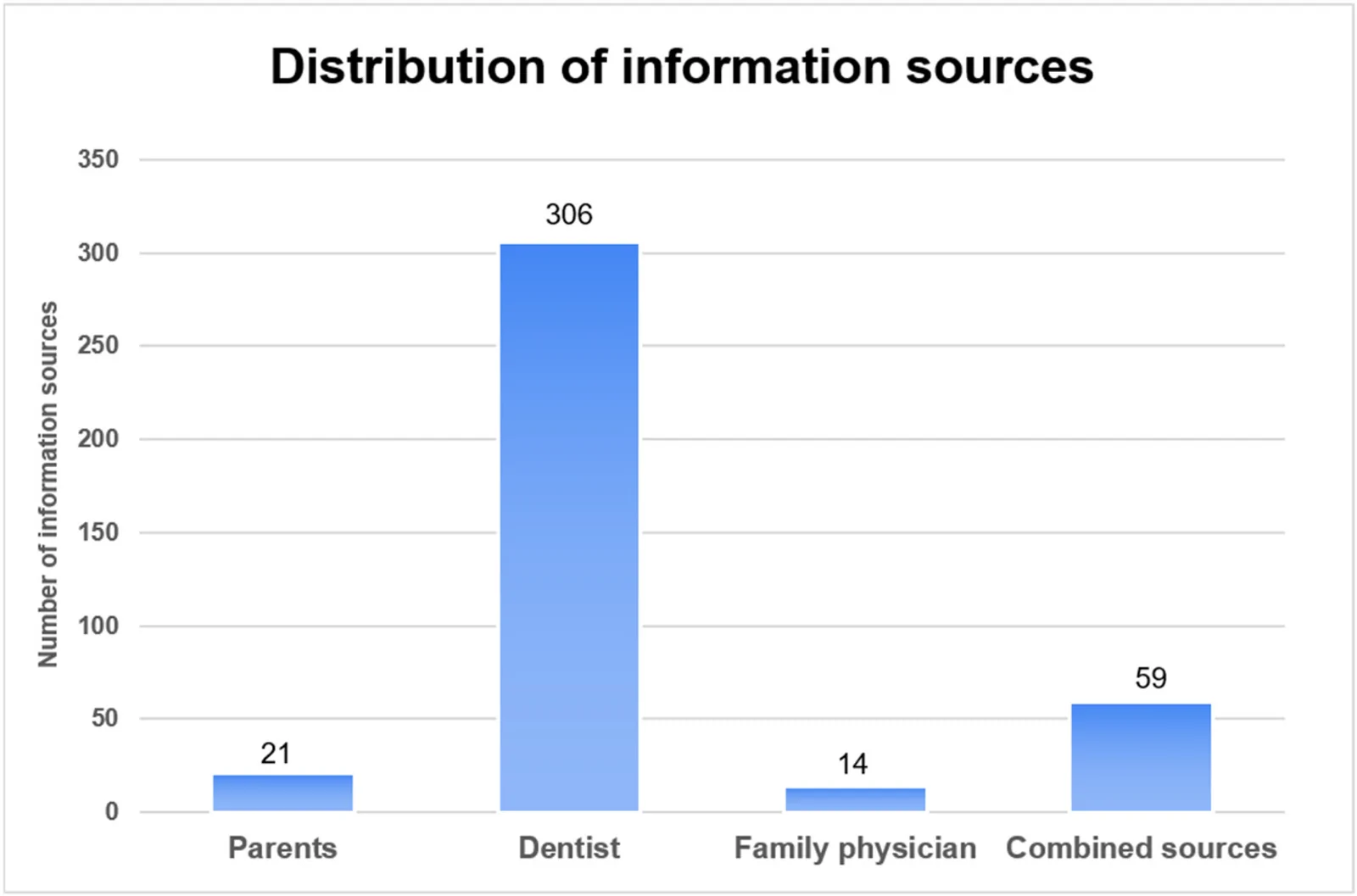
Information sources declared by the study participants.

Several sources of parental oral health information were recorded: dentists, family physicians, parents or relatives, and combined sources. As indicated in Figure 1, dentists were the main declared source of oral health information, accounting for 76.50% of responses. Family physicians were reported as an information source by 3.50% of parents, while parents or relatives accounted for 5.25%. Combined sources were reported by 14.75% of participants.

Parental information level was retained as a secondary descriptive variable because it reflects the educational context documented in the clinical records. However, because the uninformed group included only 13 participants, comparisons between informed and uninformed parents were interpreted as exploratory. In this sample, parental information level was not significantly associated with caries severity (*p* = 0.745), and this variable was not used to support the main conclusions of the study.

As indicated in Table 2, there was no statistically significant difference between the type of caries and the type of parent—informed or uninformed. The percentages in all categories were rather similar, with the highest variation of 10% recorded for incipient caries (where 25.06% children had informed parents, compared to 15.38% children with uninformed parents), as well as for advanced caries with pulpal/periapical involvement, where the percentages were reversed—38.46% children had uninformed parents, while 28.68% had informed parents. Overall, parental information level was not significantly associated with caries severity in this analysis (*p* = 0.745). Because only 13 parents were classified as uninformed, this comparison should be interpreted cautiously.

#### 3.3.2. Prenatal Variables and Lactose Intolerance

The prenatal context was analyzed with respect to documented prenatal nicotine exposure and maternal antibiotic or systemic medication use during pregnancy (Table 5). Statistically significant differences were observed between parental information groups for both prenatal exposure variables.

Prenatal nicotine exposure was more frequent in the Uninformed group than in the Informed group (92.31% versus 17.83%; *p* < 0.0005). Documented maternal antibiotic or systemic medication use during pregnancy was also more frequent in the Uninformed group (100% versus 23.26%; *p* < 0.0005). The corresponding effect-size values suggested moderate associations (ω = 0.329 and ω = 0.311, respectively).

The effect-size values were ω = 0.329 for prenatal nicotine exposure and ω = 0.311 for maternal antibiotic or systemic medication use during pregnancy. The association between parental information level and prenatal nicotine exposure showed a moderate magnitude (ω = 0.329), similar to the association with maternal antibiotic or systemic medication use during pregnancy which also showed a moderate magnitude (ω = 0.311).

Documented prenatal nicotine exposure and maternal antibiotic or systemic medication use during pregnancy were also associated with lactose intolerance in the descriptive analysis (Table 6).

Among children with documented prenatal nicotine exposure, 50 of 81 children (61.73%) had lactose intolerance, compared with 115 of 319 children (36.05%) without prenatal nicotine exposure. When analyzed from the perspective of the lactose-intolerant group, 50 of 165 children (30.30%) had prenatal nicotine exposure. Similarly, among children with documented maternal antibiotic or systemic medication use during pregnancy, 70 of 103 children (67.96%) had lactose intolerance, compared with 95 of 297 children (31.99%) without this exposure. Both associations were statistically significant (*p* < 0.0005) (Table 2). The effect-size analysis indicated a small-to-moderate association between prenatal nicotine exposure and lactose intolerance (ω = 0.210), and a moderate association between documented maternal antibiotic or systemic medication use during pregnancy and lactose intolerance (ω = 0.320).

#### 3.3.3. Parental Information Level, Oral Hygiene, and Dietary Variables

Postnatal oral health behavior was assessed through variables related to oral hygiene routines and dietary exposure.

For the 400 children and their parents included in the study sample, oral hygiene debut and habits are analyzed in Table 7.

Recorded parental information level was significantly associated with the timing of toothbrushing initiation, as the distribution of toothbrushing onset differed between the informed and uninformed groups. In the Informed group, almost a quarter of parents started brushing the primary teeth of their children at the age of 4 months (21.71%), and almost half at the age of 6 months (44.70%). Around a third of them performed this activity after 1 year of age. In contrast, in the group of uninformed participants, the onset of brushing was delayed, being initiated only at the age of 12 months (38.46%) or 24 months (61.54%). The difference between groups was statistically significant (*p* < 0.0005) (Table 8).

Among parents classified as informed, 14.47% reported that their children brushed twice daily, compared with 30.77% among parents classified as uninformed. Given the small number of Uninformed parents, the overall differences between groups were not statistically significant, *p* = 0.115 (Table 8).

With respect to the toothbrush type, both groups exhibited the same trend towards the use of the manual toothbrush, accounting for 95.35% of the Informed parents, and 100% of the Uninformed parents. The use of an electric toothbrush for their children was limited to only 4.65% of the Informed parents, and this very small percentage for the overall study group indicated no statistically significant differences between groups from this point of view. The choice of toothpaste based on the fluoride content was also a preference that seemed to be divergent between the two groups. The percentage of parents that used fluoride toothpaste for their children was rather similar for both groups, around 30%, with a slight predominance of parents in the Uninformed group. Differences between groups were also observed regarding the use of the non-fluoride toothpaste, which was declared by 68.99% of the Informed parents, and by 61.54% of the Uninformed parents, but overall, those were not statistically significant (*p* = 0.555). Toothpaste type was not significantly associated with parental information level in this analysis. The effect-size values showed that the association between parental information level and the onset of toothbrushing was small to moderate (r = 0.216), whereas the associations with daily toothbrushing frequency, toothbrush type, and toothpaste type were very weak (ω = 0.081, ω = 0.040, and ω = 0.029, respectively).

The analysis of dietary habits and risk factors in relation to the level of hygiene awareness is presented in Table 9. The duration of night feeding after the first six months of age differed significantly between the two parental information groups (*p* = 0.012). Most Informed parents stopped the nocturnal feeding between 2 years and 6 months, respectively 3 years of age (266 participants, representing 68.74% of all Informed parents). The other Informed parents were similarly divided between two different time periods: prior or equal to 2 years, and after 3 years. In contrast, in the Uninformed group of parents, night feeding was stopped mostly after 3 years of age (61.54%), and the rest after 2 years and 6 months (38.46%), while no entry was identified prior to this age. In the uninformed group, night feeding was stopped after 2 years and 6 months in 38.46% of cases and after 3 years and 6 months in 61.54% of cases.

Sweets intake frequency was also associated with recorded parental information level. Almost half of the parents classified as informed reported sweets intake less than once per day, whereas children of parents classified as uninformed had more frequent reported sweets intake. Specifically, 61.54% of children in the uninformed group were reported to consume sweets two to three times per day, while 38.46% consumed sweets once per day. The association was statistically significant (*p* < 0.0005).

The consumption of sweetened beverages was also statistically significantly different between the two study groups, with *p* < 0.0005. In this sample, all children of parents classified as uninformed were recorded as non-consumers of sweetened beverages, whereas 43.41% of children of parents classified as informed were recorded as non-consumers.

Carbonated beverage intake was very similar between groups, as most children were recorded as non-consumers (396/400, 99.0%). Only 4 participants from the Informed parents group declared that their children consumed carbonated beverages, leading to no statistically significant difference between the groups (*p* = 0.713).

Although several associations reached statistical significance, the corresponding effect sizes were small or small to moderate. The effect size was low for the duration of night feeding (r = 0.126), small to moderate for sweets intake frequency (r = 0.250), and low to moderate for sweetened beverage intake (ω = 0.202). These values indicate that the observed differences should not be overinterpreted, especially considering the small size of the uninformed group.

#### 3.3.4. Prenatal Variables and Caries Severity

Both prenatal nicotine exposure and maternal antibiotic or systemic medication use during pregnancy showed no statistically significant relationship with the distribution of carious lesions for the children included in the study sample, as indicated in Table 9.

Within the group of children with documented prenatal nicotine exposure, the distribution across the four caries severity categories was relatively balanced. A similar pattern was observed among children without documented prenatal nicotine exposure. Overall, prenatal nicotine exposure was not significantly associated with caries severity in this sample (*p* = 0.390).

The analysis of maternal antibiotic or systemic medication use during pregnancy yielded similar results, with no significant association with caries severity (*p* = 0.607). Within the exposed group, there was a slight predominance of children with advanced caries with pulpal/periapical involvement (32.04%) relative to the other categories, while in the non-exposed group, a higher percentage was recorded for the cavitated non-complicated caries (31.65%). The percentages of children from the other categories were relatively similar, with the smallest values for participants with no caries, for both exposed and non-exposed children.

The effect-size values were r = 0.043 for prenatal nicotine exposure and r = 0.026 for maternal antibiotic or systemic medication use during pregnancy. Prenatal nicotine exposure had an effect size of r = 0.043, while maternal antibiotic or systemic medication use during pregnancy had an effect size of r = 0.026.

## 4. Discussion

The present study investigated the relationship between parental oral health information, early preventive behavior, dietary habits, prenatal context, and the severity of dental caries in primary dentition. The main finding was that caries severity was more closely associated with postnatal behavioral variables than with the prenatal variables analyzed. In the univariate analyses, delayed initiation of toothbrushing, prolonged night feeding, frequent sweets intake, and sweetened beverage consumption were associated with more severe carious lesions. In the adjusted logistic regression model, only delayed toothbrushing onset and sweetened beverage consumption remained statistically significant after Bonferroni correction.

Although several variables reached statistical significance in the univariate analyses, their clinical relevance should be interpreted according to the magnitude and consistency of the associations. Some statistically significant associations had small effect sizes, such as toothbrushing onset, night feeding duration, toothbrushing frequency, toothbrush type, and toothpaste type. Therefore, these findings indicate measurable differences within the analyzed sample, but they should not be interpreted as strong individual predictors of caries severity. Greater clinical relevance was attributed to findings that remained significant in the multivariable model and were consistent with established preventive principles, particularly delayed toothbrushing onset and sweetened beverage intake. Sweetened beverage intake showed the strongest association in the adjusted model, whereas delayed toothbrushing onset is clinically relevant because it reflects the timing of preventive behavior during the early eruption period of primary teeth. Thus, the clinical interpretation of the results was based not only on *p*-values, but also on effect size, adjusted analysis, biological plausibility, and practical relevance for preventive counseling.

Although child age and sex were not significantly associated with caries severity in the univariate analyses, they were further evaluated as potential confounders in a sensitivity logistic regression model. Their inclusion did not materially alter the association pattern observed in the primary behavioral model. This supports the interpretation that, within the restricted 3–6-year age interval of this clinical sample, the main associations were more closely related to oral hygiene and dietary behaviors than to basic demographic variables. Nevertheless, the limited age range and clinical-record-based design restrict broader conclusions regarding age- or sex-specific patterns.

From a clinical perspective, these findings indicate that parental knowledge alone is not sufficient to reduce caries severity if it is not translated into consistent daily practices. Preventive counseling should therefore move beyond general information and provide parents with specific, repeated behavioral instructions. These should include when to start toothbrushing, how often brushing should be performed, the need for caregiver supervision, appropriate use of fluoridated toothpaste, reduction in sweetened beverages, and practical alternatives for bedtime and between-meal drinks. Repeated reinforcement of these instructions during pediatric dental visits may be particularly important for children who already show early carious lesions, because these children may benefit from closer monitoring and more structured home-based prevention.

### Secondary Exploratory Findings

Prenatal nicotine exposure, maternal antibiotic or systemic medication use during pregnancy, lactose intolerance, and parental information level were treated as secondary exploratory variables. Prenatal nicotine exposure and maternal medication/systemic drug use were not significantly associated with caries severity, and therefore they did not drive the main conclusion of the study. Lactose intolerance was associated with some lesion-count outcomes, but the clinical records did not provide detailed information on dietary substitutions, lactose-free products, added sugars, feeding frequency, or bedtime intake. For this reason, lactose intolerance should be interpreted as an exploratory clinical-record variable rather than as an independent dietary determinant of caries severity.

The present study investigated the relationship between parental oral health information, early preventive behavior, dietary habits, prenatal context, and the severity of dental caries in primary dentition. The main finding was that caries severity was more closely associated with postnatal behavioral variables than with the prenatal variables analyzed. In the univariate analyses, delayed initiation of toothbrushing, prolonged night feeding, frequent sweets intake, and sweetened beverage consumption were associated with more severe carious lesions. In the adjusted logistic regression model, only delayed toothbrushing onset and sweetened beverage consumption remained statistically significant after Bonferroni correction. Similarly, the analysis of lesion number indicated that caries burden was mainly associated with modifiable postnatal factors, particularly hygiene-related and dietary variables. This finding is consistent with the multivariable model, in which delayed toothbrushing onset and sweetened beverage intake remained associated with higher odds of major carious lesions. By contrast, prenatal exposure to nicotine and maternal antibiotic or systemic medication use during pregnancy did not show a statistically significant association with the distribution of caries severity in this sample.

By contrast, prenatal exposure to nicotine and maternal antibiotic or systemic medication use during pregnancy did not show a statistically significant association with the distribution of caries severity in this sample. These findings support the current interpretation of early childhood caries as a multifactorial disease in which biological susceptibility is shaped by modifiable family-level behaviors, especially plaque control, fluoride exposure, sugar frequency, feeding routines, and parental implementation of preventive advice [14,15,16,17,18,19,20,21].

Age and sex were not statistically significantly associated with caries severity in the present sample. This finding suggests that, within the restricted 3–6-year age interval analyzed here, differences in caries severity were more closely related to modifiable oral hygiene and dietary behaviors than to basic demographic variables. However, the absence of statistical significance should be interpreted cautiously, because the study was not designed to investigate age- or sex-specific biological mechanisms.

The high proportion of children with carious lesions in the present group confirms the persistent burden of caries in primary dentition. Only 69 children were caries-free, while the remaining children presented incipient, cavitated non-complicated, or advanced lesions with pulpal/periapical involvement. This distribution is clinically relevant because untreated caries in primary teeth is not a transient condition without consequences. Recent pediatric dentistry literature emphasizes that early childhood caries may affect mastication, sleep, growth, school attendance, quality of life, and the risk of caries in permanent dentition [14,15,19,20]. The present results therefore have preventive importance beyond the primary dentition itself. The identification of modifiable factors associated with severe caries may help shift clinical management from late restorative treatment toward early, risk-based prevention [18,20,21].

A first relevant aspect concerns the relationship between parental information and clinical outcomes. In this study, most caregivers were classified as informed regarding the chronology of oral hygiene, and the dentist was the main declared source of oral health information. However, parental information level as a binary variable was not significantly associated with caries severity. This result should not be interpreted as evidence that parental education is irrelevant. Rather, it suggests that declared knowledge may not be sufficient unless it is converted into stable daily practices. Recent studies on parental knowledge, oral health literacy, self-efficacy, and early childhood caries have shown that the effect of parental education is mediated by behavior, including supervised toothbrushing, use of fluoridated toothpaste, dietary control, preventive attendance, and the capacity to maintain routines in the home environment [22,23,24,25,26,27,28,29,30,31].

Parental information level should therefore be interpreted with caution in the present study. Although it was associated with several reported behaviors in descriptive analyses, the variable was defined using a narrow clinical-record criterion and the uninformed group was small. Therefore, parental information level should not be considered a central independent predictor of caries severity in this dataset. The adjusted findings support a stronger role for specific modifiable behaviors, particularly delayed toothbrushing onset and sweetened beverage intake.

This distinction between knowledge and practice is important for interpreting the present findings. A parent may know that oral hygiene should start early but may still delay brushing, use a non-fluoridated toothpaste, allow frequent sugar exposure, or maintain nocturnal feeding for a prolonged period. This knowledge-practice gap has been reported in several recent studies, including Romanian data on parental knowledge, attitudes, and practices regarding early childhood caries [22,27,31]. In the present study, the dentist was the dominant information source, which confirms the central role of dental professionals in prevention. Nevertheless, the persistence of severe caries among children of informed parents indicates that clinical counseling should be practical and repeated, not limited to general explanations. Preventive communication should include clear instructions on when brushing begins, how much toothpaste is used, how toothbrushing is supervised, which drinks should be avoided, and when nocturnal feeding should be reduced after eruption of primary teeth.

The Romanian context also supports this interpretation. Recent national and regional studies have reported a high prevalence of caries and an association between oral health outcomes and parental education, socioeconomic variables, dietary habits, dental attendance, and preventive behavior [32,33,34,35,36,37]. These findings are consistent with the present study, although the current sample focuses on younger children and primary dentition. The local relevance of the results is important because oral health behaviors are shaped by family routines, access to pediatric dentistry, cultural norms regarding feeding, and the perceived importance of primary teeth. In populations where dental attendance is often symptom-driven rather than preventive, parental counseling must begin before pain, infection, or advanced caries with pulpal/periapical involvement become the reason for consultation [34,35,36,37].

The onset of toothbrushing was one of the strongest behavioral findings. Children whose toothbrushing began later, particularly at 24 months, had a higher proportion of advanced lesions with pulpal/periapical involvement and a lower proportion of caries-free status. This association is clinically plausible. The first erupted primary teeth are exposed to dental biofilm soon after eruption, and delayed plaque control leaves immature enamel vulnerable to repeated demineralization. Recent reviews and clinical recommendations support toothbrushing from eruption of the first primary tooth, using age-appropriate amounts of fluoridated toothpaste and parental supervision [14,17,18,19,20,21]. The present findings are also aligned with recent observational studies reporting higher caries prevalence among children with irregular or insufficient brushing habits [38,39,40,41,42,43,44,45].

The type of toothbrush showed a statistically significant association with caries severity in the univariate analysis. Children using electric toothbrushes had a higher proportion of caries-free status and fewer advanced lesions with pulpal/periapical involvement. This result should be interpreted cautiously because only a small number of children used electric toothbrushes. The association may reflect not only the mechanical effect of the device, but also higher parental involvement, better socioeconomic status, more structured routines, or greater interest in oral hygiene. Recent literature suggests that toothbrushing effectiveness depends less on the device alone and more on frequency, technique, duration, fluoride exposure, and supervision [20,21,45,46,47,48,49,50,51,52,53,54,55,56,57,58,59,60,61,62,63,64,65]. Therefore, the present finding should be presented as an association, not as proof that electric toothbrushes independently reduced caries severity.

The toothpaste variable also deserves careful interpretation. Children using non-fluoridated toothpaste had higher proportions of cavitated non-complicated and advanced caries with pulpal/periapical involvement, while the group using fluoridated toothpaste showed a more favorable distribution. This is compatible with strong evidence supporting fluoride toothpaste for caries prevention [17,20,59]. The preventive effect of fluoride depends on regular exposure, adequate concentration, brushing frequency, and the amount used. In young children, the benefit must be balanced with supervision to reduce excessive ingestion, but current pediatric recommendations support age-appropriate fluoridated toothpaste rather than fluoride avoidance [17,20,59,60]. The present data suggest that toothpaste choice may represent both a direct preventive factor and a marker of parental preventive orientation.

Dietary variables were strongly associated with caries severity. Frequent sweets intake and sweetened beverage consumption were linked to more severe lesions. This supports the established cariological model in which repeated exposure to fermentable carbohydrates maintains low plaque pH, favors acidogenic and aciduric bacterial communities, and increases the cumulative time spent under the critical pH threshold. Recent studies and reviews confirm that sugar exposure remains one of the most consistent modifiable determinants of dental caries in children [46,47,48,49,50,51,52]. The present results are especially relevant because the difference was not limited to caries presence, but extended to severity. This distinction matters clinically: frequent sugar exposure does not merely increase the chance of detecting a lesion; it may also contribute to progression toward cavitated non-complicated and advanced caries with pulpal/periapical involvement when combined with insufficient hygiene and inadequate fluoride exposure.

Sweetened beverages were particularly important. Children who consumed sweetened beverages had a higher proportion of cavitated non-complicated and advanced caries with pulpal/periapical involvement compared with children who did not consume them. This is consistent with recent literature showing that sugar-containing drinks contribute to early sugar exposure and may become embedded in daily routines from infancy or toddlerhood [44,47,48,49,50,51,52]. Liquid sugars are relevant because they are easy to consume frequently, may be offered between meals, and can be used during comfort routines. Their cariogenic potential increases when intake occurs outside structured meals or near bedtime, when salivary clearance is reduced [41,47,48,49,50,51,52].

The distribution of sweetened beverage intake according to parental information level should be interpreted cautiously. In this sample, all children of uninformed parents were recorded as non-consumers of sweetened beverages, whereas more than half of the children of informed parents had documented intake. This unexpected pattern may reflect the very small size of the uninformed group, differences in reporting accuracy, or the limitations of retrospective clinical records. Therefore, it should not be interpreted as evidence that uninformed parents had more favorable dietary practices.

In the present study, carbonated beverages were not significantly associated with caries severity, but the number of exposed children was very small. Therefore, the lack of significance should not be interpreted as evidence of safety; it is more likely a consequence of low exposure frequency and limited statistical power.

Night feeding duration was significantly associated with caries severity. Children with longer night feeding tended to present more severe lesions. This finding is biologically plausible because nocturnal feeding may prolong carbohydrate exposure during a period of reduced salivary flow and limited oral clearance. The literature on breastfeeding and caries has become more nuanced in recent years. Breastfeeding itself has multiple health benefits and should not be presented as inherently harmful. However, prolonged or frequent nocturnal feeding after tooth eruption, especially when combined with poor oral hygiene and additional sugar exposure, may increase caries risk [30,43,53,54,55,56,57,58]. The clinical message should therefore avoid discouraging breastfeeding in general. The appropriate interpretation is that feeding counseling must be integrated with eruption status, oral hygiene onset, fluoride exposure, and bedtime routines.

The prenatal variables analyzed in this study showed a different pattern. Prenatal nicotine exposure and maternal antibiotic or systemic medication use during pregnancy were associated with parental information level and lactose intolerance in the descriptive analyses, but they were not significantly associated with caries severity. This finding should be interpreted cautiously. Recent systematic reviews suggest that maternal smoking during pregnancy and passive smoke exposure may be associated with childhood caries, but these associations are susceptible to confounding by socioeconomic status, parental education, dietary patterns, oral hygiene, and access to care [66,67,68,69,70]. The absence of a significant relationship in the present sample does not exclude a prenatal contribution to oral health vulnerability. It indicates that, within this dataset, postnatal behaviors were more clearly associated with the severity of carious lesions.

Lactose intolerance was retained only as a hypothesis-generating clinical-record observation. Although it was associated with some lesion-count outcomes, this finding is difficult to interpret biologically because the available records did not provide detailed information on the diagnostic criteria for lactose intolerance, age at diagnosis, symptom severity, dietary substitutions, lactose-free products, added sugars, feeding frequency, or bedtime intake. Therefore, no mechanistic interpretation can be made from these data. The observed association should not be interpreted as evidence that lactose intolerance directly influences caries development or severity. It indicates only that, in this clinical-record-based sample, documented lactose intolerance co-occurred with a higher number of some carious lesion categories. This observation requires confirmation in future prospective studies using structured dietary assessment.

The antibiotic/drug exposure variable also requires cautious interpretation. In its present form, it combines potentially different exposures, including antibiotics prescribed during pregnancy and other medication use. Antibiotics administered for a medical indication should not be discussed as inappropriate exposure unless the dataset distinguishes prescribed treatment from self-medication, class of antibiotic, trimester, dose, duration, and indication. Recent evidence on antibiotic exposure and dental health focuses mainly on early childhood exposure and dental developmental defects, staining, or caries outcomes, but causal interpretation remains difficult because infections, fever, medication class, timing, and confounding behaviors may all influence oral outcomes [68]. For the present manuscript, this variable should be discussed as an exploratory prenatal/anamnestic factor, not as a causal determinant of caries severity.

The logistic regression model adds value because it evaluates several behavioral factors simultaneously. In the adjusted model, delayed toothbrushing onset and sweetened beverage consumption remained independently associated with higher odds of major caries after Bonferroni correction. Daily toothbrushing frequency and sweets intake frequency showed clinically relevant trends, but they did not remain statistically significant under the conservative Bonferroni threshold. Therefore, these variables should be interpreted cautiously as relevant behavioral indicators rather than as independent factors in the adjusted model [18,21,38,39,61].

The present study has limitations that should be considered when interpreting the findings. The cross-sectional design does not allow temporal or causal inference, so the reported results should be understood as associations observed within this sample. The retrospective, clinical-record-based design also means that the analysis depended on the completeness and accuracy of information documented during routine pediatric dental assessment. Behavioral and dietary variables were originally reported by parents or legal guardians during clinical history taking and may have been affected by recall bias or social desirability bias.

Because the study population was derived from pediatric dental records of children attending clinical services, referral bias and care-seeking bias should also be considered. Children who are brought to dental clinics may differ from the general pediatric population in terms of oral symptoms, parental concern, previous dental experience, access to care, socioeconomic background, and perceived treatment need. Therefore, the high proportion of children with carious lesions in this sample may partly reflect the clinical-care-seeking nature of the population rather than the true prevalence of caries in the wider community. Consequently, the findings should not be interpreted as population prevalence estimates and should be generalized with caution outside similar clinical settings. The observed associations between oral hygiene, dietary behaviors, and caries severity are most applicable to children presenting for pediatric dental assessment, while community-based studies would be needed to confirm whether the same patterns apply to the general pediatric population.

The included children were aged between 3 and 6 years, and age and sex were available for analysis. Neither variable was statistically significantly associated with caries severity in this sample. Nevertheless, the narrow age range limits the ability to detect broader age-related patterns, and the clinical-record-based design limits interpretation of sex-specific differences. Other potentially relevant confounders, including socioeconomic status, parental education level, household income, place of residence, access to dental care, previous preventive interventions, and professional fluoride exposure, were not available in a standardized form and could not be included in the statistical models.

Parental information level was classified according to information documented in the clinical files, not through a validated questionnaire. The marked imbalance between the informed and uninformed groups, with only 13 parents classified as uninformed, limits the interpretation of comparisons based on this variable. Caries classification also reflected the diagnostic detail available in the original clinical records. Records with insufficient information for severity classification were excluded to reduce misclassification, but no additional examinations were performed for research purposes.

Maternal antibiotic or systemic medication use during pregnancy was recorded as a broad exploratory variable. The available records did not provide systematic information on medication class, indication, trimester of exposure, dose, duration, or maternal medical condition, so this variable should not be interpreted pharmacologically. Lactose intolerance was also analyzed as an exploratory clinical-record variable, and the absence of detailed dietary information limits interpretation of its association with lesion counts.

Despite these limitations, the findings are clinically relevant because they point to modifiable postnatal behaviors that can be addressed during early dental counseling. Early initiation of toothbrushing, twice-daily supervised brushing, appropriate use of fluoridated toothpaste, reduced frequency of sugar exposure, avoidance of sweetened beverages, and counseling regarding prolonged night feeding should remain central components of preventive care in children with primary dentition [20,21,22,23,24,25,28,29,30,31,58,59,60,61,62,63,64,65,66,67,68,69,70,71,72].

The parental information variable should be interpreted with caution because only 13 participants were classified as uninformed. This imbalance limits the reliability of direct comparisons between informed and uninformed caregivers. Nevertheless, the variable was retained because it provides useful clinical context regarding parental oral health information and care-related behavior. The findings related to parental information level should therefore be considered descriptive and exploratory, whereas the main conclusions are based on the primary behavioral variables retained in the adjusted model, especially toothbrushing onset and sweetened beverage intake.

Model calibration and multicollinearity diagnostics were not included in the present analysis; therefore, the logistic regression findings should be interpreted as exploratory adjusted associations.

## 5. Conclusions

In this cross-sectional clinical-record-based study, major carious lesions in primary dentition were mainly associated with modifiable postnatal behaviors. Delayed toothbrushing onset and sweetened beverage consumption remained the main independent factors associated with higher odds of major carious lesions. These findings support early, specific, and repeated parental counseling focused on supervised toothbrushing from the eruption of the first primary tooth, appropriate use of fluoridated toothpaste, and avoidance of sweetened beverages. The results should be interpreted as associations rather than causal relationships.

## Figures and Tables

**Table 1:** Distribution of the carious lesions according to hygiene levels.

Parameter	Category	Carious Lesions				Total	*p*
		None	Incipient	Cavitated Non-Complicated	Complicated		
		69 (100%)	99 (100%)	116 (100%)	116 (100%)		
Age at toothbrushing onset	4 months	14 (16.67%)	19 (22.62%)	27 (32.14%)	24 (28.57%)	84 (100%)	0.038 *# τ_b_ = 0.088
	6 months	37 (21.39%)	49 (28.32%)	44 (25.43%)	43 (24.86%)	173 (100%)	
	12 months	13 (23.21%)	11 (19.64%)	19 (33.93%)	13 (23.21%)	56 (100%)	
	24 months	5 (5.75%)	20 (22.99%)	26 (29.89%)	36 (41.38%)	87 (100%)	
Frequency of daily toothbrushing	1 time/day	53 (15.59%)	79 (23.24%)	99 (29.12%)	109 (32.06%)	340 (100%)	0.005 ** r = 0.181
	2 times/day	16 (26.67%)	20 (33.33%)	17 (28.33%)	7 (11.67%)	60 (100%)	
Type of toothbrush (toothbrush)	Manual	62 (16.23%)	93 (24.35%)	113 (29.58%)	114 (29.84%)	382 (100%)	0.005 **# *r* = 0.139
	Electric	7 (38.89%)	6 (33.33%)	3 (16.67%)	2 (11.11%)	18 (100%)	
Type of toothpaste used	Fluoride	30 (24.00%)	34 (27.20%)	28 (22.40%)	33 (26.40%)	125 (100%)	0.025 **# *r* = 0.112
	Non-fluoridated	39 (14.18%)	65 (23.64%)	88 (32.00%)	83 (30.18%)	275 (100%)	

* Jonckheere-Terpstra test. ** Mann–Whitney U test. # Statistically significant.

**Table 2:** Distribution of the carious lesions according to the postnatal context.

Parameter	Category			Carious Lesions		Total	*p*
		None	Incipient	Cavitated Non-Complicated	Complicated		
		69 (100%)	99 (100%)	116 (100%)	116 (100%)		
Duration of night feeding (post-6 months)	1 year and 6 months	1 (5.00%)	5 (25.00%)	7 (35.00%)	7 (35.00%)	20 (100%)	0.032 *# τ_b_ = 0.090
	2 years	19 (45.24%)	9 (21.43%)	6 (14.29%)	8 (19.05%)	42 (100%)	
	2 years and 6 months	21 (15.22%)	36 (26.09%)	49 (35.51%)	32 (23.19%)	138 (100%)	
	3 years	17 (12.78%)	32 (24.06%)	35 (26.32%)	49 (36.84%)	133 (100%)	
	3 years and 6 months	11 (16.42%)	17 (25.37%)	19 (28.36%)	20 (29.85%)	67 (100%)	
Recorded parental information level	Informed	66 (17.05%)	97 (25.06%)	113 (29.2%)	111 (28.68%)	387 (100%)	0.745 ** r = 0.016
	Uninformed	3 (23.08%)	2 (15.38%)	3 (23.08%)	5 (38.46%)	13 (100%)	
Frequency of sweets intake	2–3 times/day	1 (4.55%)	7 (31.82%)	8 (36.36%)	6 (27.27%)	22 (100%)	<0.0005 *# τ_b_ = 0.208
	1 time/day	32 (15.69%)	53 (25.98%)	62 (30.39%)	57 (27.94%)	204 (100%)	
	Several times/week	20 (15.15%)	22 (16.67%)	38 (28.79%)	52 (39.39%)	132 (100%)	
	Rarely	16 (38.1%)	17 (40.48%)	8 (19.05%)	1 (2.38%)	42 (100%)	
Intake of sweetened beverages	Yes	28 (12.79%)	39 (17.81%)	74 (33.79%)	78 (35.62%)	219 (100%)	<0.0005 **# *r* = 0.234
	No	41 (22.65%)	60 (33.15%)	42 (23.2%)	38 (20.99%)	181 (100%)	
Carbohydrate intake of carbona-ted beverages	Yes	0 (0%)	2 (50%)	0 (0%)	2 (50%)	4 (100%)	0.586 ** r = 0.027
	No	69 (17.42%)	97 (24.49%)	116 (29.29%)	114 (28.79%)	396 (100%)	

* Jonckheere-Terpstra test. ** Mann-Whitney U test. # Statistically significant.

**Table 3:** Binary logistic regression parameters predicting the likelihood of major carious lesions based on oral hygiene and dietary factors.

Parameter	B ^1^	Sig	Exp (B)	CI Interval for Exp (B)	
				Lower	Upper
Toothbrushing onset	0.381	0.002	1.464	1.148	1.866
Night feeding	−0.025	0.811	0.975	0.793	1.199
Frequency of daily toothbrushing	−0.653	0.036	0.520	0.282	0.959
Toothbrush type	1.062	0.064	2.892	0.942	8.880
Toothpaste type	0.027	0.916	1.027	0.622	1.696
Frequency of sweets intake	0.244	0.027	1.276	1.028	1.584
Sweetened beverages	0.911	<0.0005	2.488	1.561	3.963
Carbonated beverages	−0.773	0.449	0.481	0.062	3.419

^1^ B coefficients indicate the direction and magnitude of association with major carious lesions. Exp (B) indicates the change in the odds of major carious lesions for each one-category increase in ordinal variables or for the coded category in binary variables. The outcome was coded as minor caries versus major caries. Minor caries included absent and incipient lesions, whereas major caries included cavitated non-complicated caries and advanced caries with pulpal/periapical involvement. Ordinal variables were coded in ascending risk-related order: toothbrushing onset from earlier to later initiation, night feeding duration from shorter to longer duration, and sweets intake frequency from lower to higher frequency. Binary variables were coded as follows: toothbrushing frequency, twice daily versus once daily; toothbrush type, manual versus electric; toothpaste type, non-fluoridated or unknown-fluoride toothpaste versus fluoridated toothpaste; sweetened beverage intake, yes versus no; and carbonated beverage intake, yes versus no. Sig represents the statistical significance of the test.

**Table 4:** Distribution of the number of carious lesions according to oral hygiene, dietary, and exploratory variables.

Parameter	Category	Number of Carious Lesions					
		Incipient		Cavitated Non-Complicated		Advanced with Pulpal/Periapical Involvement	
		Median/Mean ± SD	*p*	Median/Mean ± SD	*p*	Median/Mean ± SD	*p*
Recorded parental information level	Informed	3/2.5 ± 1.6	0.918 *	2/1.7 ± 1.6	0.289 *	0/1.2 ± 1.8	0.389 *
	Uninformed	3/2.4 ± 1.5	*r* = 0.005	3/2.2 ± 1.9	*r* = 0.053	0/1.6 ± 2.1	*r* = 0.043
Prenatal exposure to toxic substances (nicotine)	Yes	3/2.5 ± 1.5	0.643 *	2/1.7 ± 1.6	0.915 *	0/1.1 ± 1.8	0.602 *
	No	3/2.6 ± 1.6	*r* = 0.023	2/1.7 ± 1.6	*r* = 0.005	0/1.2 ± 1.9	*r* = 0.026
Maternal antibiotic or systemic medication use during pregnancy	Yes	2/2.3 ± 1.6	0.064 *	2/1.5 ± 1.5	0.099 *	0/1.4 ± 2	0.182 *
	No	3/2.6 ± 1.5	*r* = 0.093	2/1.8 ± 1.6	*r* = 0.082	0/1.1 ± 1.8	*r* = 0.067
Age at toothbrushing onset	4 months	3/2.7 ± 1.7		2/1.9 ± 1.7		0/1.1 ± 1.8	
	6 months	3/2.4 ± 1.5	0.433 **	1/1.5 ± 1.6	0.067 **	0/1 ± 1.8	0.039 **#
	12 months	3/2.5 ± 1.8	η^2^ = 0.001	2/1.6 ± 1.6	η^2^ = 0.010	0/1.1 ± 1.8	η^2^ = 0.014
	24 months	3/2.7 ± 1.3		2/2.1 ± 1.6		0/1.7 ± 2	
Duration of night feeding (post-6 months)	1 year and 6 months	2/2.9 ± 1.6		2/2 ± 1.5		0/1.6 ± 2	
	2 years	2/1.6 ± 1.7	0.002 **#	0/0.8 ± 1.3	0.004 **#	0/1 ± 1.8	0.079 **
	2 years and 6 months	3/2.7 ± 1.6	η^2^ = 0.031	2/1.8 ± 1.7	η^2^ = 0.029	0/0.9 ± 1.7	η^2^ = 0.011
	3 years	3/2.6 ± 1.5		2/1.8 ± 1.6		0/1.5 ± 2	
	3 years and 6 months	3/2.5 ± 1.5		3/1.9 ± 1.7		0/1.2 ± 1.8	
Lactose intolerance	Yes	3/2.4 ± 1.4	0.318 *	2/2 ± 1.5	0.016 *#	0/1.5 ± 2	0.001 *#
	No	3/2.6 ± 1.7	*r* = 0.050	1/1.6 ± 1.7	*r* = 0.120	0/0.9 ± 1.7	*r* = 0.162
Frequency of daily toothbrushing	1 time/day	3/2.6 ± 1.5	0.479 *#	2/1.8 ± 1.6	0.081 *#	0/1.3 ± 1.9	0.001 *
	2 times/day	2/2.5 ± 1.9	*r* = 0.035	0/1.4 ± 1.7	*r* = 0.087	0/0.5 ± 1.3	*r* = 0.167
Type of sanitizing device (toothbrush)	Manual	3/2.6 ± 1.6	0.162 *	2/1.8 ± 1.6	0.026 *#	0/1.2 ± 1.9	0.082 *
	Electric	3/1.9 ± 1.6	*r* = 0.070	0/0.9 ± 1.6	*r* = 0.111	0/0.4 ± 1.3	*r* = 0.087
Type of toothpaste used	With fluor	3/2.4 ± 1.7	0.459 *	1/1.4 ± 1.5	0.009 *#	0/1.2 ± 1.9	0.741 *
	Without fluor	3/2.6 ± 1.5	*r* = 0.037	2/1.9 ± 1.6	*r* = 0.131	0/1.2 ± 1.8	*r* = 0.017
Frequency of sweets intake	2–3 times/day	3/3 ± 1.3		3/2.2 ± 1.8		0/1.3 ± 2	
	1 time/day	3/2.6 ± 1.6	0.191 **	2/1.8 ± 1.6	<0.0005 **#	0/1.1 ± 1.8	<0.0005 **#
	Several times/week	3/2.4 ± 1.4	η^2^ = 0.002	2/1.9 ± 1.5	η^2^ = 0.049	0/1.6 ± 2	η^2^ = 0.045
	Rarely	2/2.1 ± 2		0/0.6 ± 1.4		0/0.1 ± 0.6	
Intake of sweetened beverages	Yes	3.5/3.5 ± 1.3	0.281 *	1/1 ± 1.2	<0.0005 *#	1.5/2 ± 2.4	0.005 *#
	No	3/2.5 ± 1.6	*r* = 0.054	2/1.7 ± 1.6	*r* = 0.232	0/1.2 ± 1.8	*r* = 0.140
Carbohydrate intake of carbonated beverages	Yes	3/2.5 ± 1.5	0.215 *	2/2.1 ± 1.6	0.334 *	0/1.4 ± 1.9	0.379 *
	No	3/2.6 ± 1.7	*r* = 0.062	0/1.3 ± 1.6	*r* = 0.048	0/0.9 ± 1.7	*r* = 0.044

* Mann–Whitney U test. ** Kruskal–Wallis H test. # Statistically significant.

**Table 5:** Distribution of the study groups according to the prenatal context.

Parameter	Category	Recorded Parental Information Level		Total	*p*
		Informed Group	Uninformed Group		
		387 Parents	13 Parents		
Prenatal exposure to toxic substances (nicotine)	Yes	69 (85.19%)	12 (14.81%)	81 (100%)	<0.0005 *# ω = 0.329
	No	318 (99.69%)	1 (0.31%)	319 (100%)	
Maternal antibiotic or systemic medication use during pregnancy	Yes	90 (87.38%)	13 (12.62%)	103 (100%)	<0.0005 *# ω = 0.311
	No	297 (100%)	0 (0%)	297 (100%)	

* Fisher’s Exact test. # Statistically significant.

**Table 6:** Distribution of the study groups according to the lactose tolerance/intolerance and the prenatal anamnestic variables.

Parameter	Category	Lactose Intolerance		Total	*p*
		Yes	No		
		165 Children	235 Children		
Prenatal exposure to toxic substances (nicotine)	Yes	50 (61.73%)	31 (38.27%)	81 (100%)	<0.0005 *# ω = 0.210
	No	115 (36.05%)	204 (63.95%)	319 (100%)	
Documented maternal antibiotic or systemic medication use during pregnancy	Yes	70 (67.96%)	33 (32.04%)	103 (100%)	<0.0005 *# ω = 0.320
	No	95 (31.99%)	202 (68.01%)	297 (100%)	

* Chi-Square test. # Statistically significant.

**Table 7:** Distribution of study groups according to the prophylactic habits (toothbrush, toothpaste).

Parameter	Category	Recorded Parental Information Level		Total	*p*
		Informed Group	Uninformed Group		
		387 Parents	13 Parents		
Age at toothbrushing onset	4 months	84 (100%)	0 (0%)	84 (100%)	<0.0005 *# *r* = 0.216
	6 months	173 (100%)	0 (0%)	173 (100%)	
	12 months	51 (91.07%)	5 (8.93%)	56 (100%)	
	24 months	79 (90.8%)	8 (9.2%)	87 (100%)	
Frequency of daily toothbrushing	1 time/day	331 (97.35%)	9 (2.65%)	340 (100%)	0.115 ** ω = 0.081
	2 times/day	56 (93.33%)	4 (6.67%)	60 (100%)	
Type of toothbrush (toothbrush)	Manual	369 (96.6%)	13 (3.4%)	382 (100%)	0.426 ** ω = 0.040
	Electric	18 (100%)	0 (0%)	18 (100%)	
Type of toothpaste used	Fluoride	120 (96.00%)	5 (4.00%)	125 (100%)	0.555 ** ω = 0.029
	Non-fluoridated	267 (97.09%)	8 (2.91%)		

* Mann–Whitney U test. ** Fisher’s Exact test. # Statistically significant.

**Table 8:** Distribution of the study groups according to dietary habits.

Parameter	Category	Recorded Parental Information Level		Total	*p*
		Informed Group	Uninformed Group		
		387 Parents	13 Parents		
Duration of night feeding (post-6 months)	1 year and 6 months	20 (100%)	0 (0%)	20 (100%)	0.012 *# *r* = 0.126
	2 years	42 (100%)	0 (0%)	42 (100%)	
	2 years and 6 months	133 (96.38%)	5 (3.62%)	138 (100%)	
	3 years	133 (100%)	0 (0%)	133 (100%)	
	3 years and 6 months	59 (88.06%)	8 (11.94%)	67 (100%)	
Frequency of sweets intake	2–3 times/day	14 (63.64%)	8 (36.36%)	22 (100%)	<0.0005 *# *r* = 0.250
	1 time/day	199 (97.55%)	5 (2.45%)	204 (100%)	
	Several times/week	132 (100%)	0 (0%)	132 (100%)	
	Rarely	42 (100%)	0 (0%)	42 (100%)	
Intake of sweetened beverages	Yes	219 (100%)	0 (0%)	219 (100%)	<0.0005 **# ω = 0.202
	No	168 (92.82%)	13 (7.18%)	181 (100%)	
Carbohydrate intake of carbonated beverages	Yes	4 (100%)	0 (0%)	4 (100%)	0.713 *** ω = 0.018
	No	383 (96.72%)	13 (3.28%)	396 (100%)	

* Mann–Whitney U test. ** Chi-Square test. *** Fisher’s Exact test. # Statistically significant.

**Table 9:** Distribution of the carious lesions according to the prenatal context.

Parameter	Category	Carious Lesions				Total	*p* *
		None	Incipient	Cavitated Non-Complicated	Complicated		
		69 (100%)	99 (100%)	116 (100%)	116 (100%)		
Prenatal exposure to toxic substances (nicotine)	Yes	16 (19.75%)	22 (27.16%)	21 (25.93%)	22 (27.16%)	81 (100%)	0.390 *r* = 0.043
	No	53 (16.61%)	77 (24.14%)	95 (29.78%)	94 (29.47%)	319 (100%)	
Maternal antibiotic or systemic medication use during pregnancy	Yes	22 (21.36%)	26 (25.24%)	22 (21.36%)	33 (32.04%)	103 (100%)	0.607 *r* = 0.026
	No	47 (15.82%)	73 (24.58%)	94 (31.65%)	83 (27.95%)	297 (100%)	

* Mann–Whitney U test.

## Data Availability

The data presented in this study are available on request from the corresponding author due to privacy, legal, and ethical restrictions.
